# Survival and glycemic control in patients with coexisting lymphoma and diabetes: a case–control analysis

**DOI:** 10.2144/fsoa-2020-0100

**Published:** 2020-10-27

**Authors:** Bria J Rice, Matthew R Buras, Heidi E Kosiorek, Kyle E Coppola, Shailja B Amin, Patricia M Verona, Curtiss B Cook, Nina J Karlin

**Affiliations:** 1 Department of Internal Medicine, Mayo Clinic, Scottsdale, AZ 85259, USA; 2 Department of Biostatistics, Mayo Clinic, Scottsdale, AZ 85259, USA; 3 Mayo Clinic Cancer Center, Mayo Clinic, Scottsdale, AZ 85259, USA; 4 Division of Hematology & Medical Oncology, Mayo Clinic Hospital, Phoenix, AZ 85054, USA; 5 Department of Information Technology, Mayo Clinic, Scottsdale, AZ 85259, USA; 6 Division of Endocrinology, Mayo Clinic, Scottsdale, AZ 85259, USA

**Keywords:** cancer, diabetes, endocrinology, lymphoma, outcomes research, survival

## Abstract

**Aim:**

We examined the effect of diabetes on survival in patients with lymphoma and the effect of lymphoma on glycemic control.

**Patients & methods:**

Patients with lymphoma with and without diabetes (2005–2016) were retrospectively identified and matched 1:1. Overall survival and progression-free survival were estimated by the Kaplan–Meier method. Hemoglobin A_1c_ (HbA_1c_) and glucose levels during the year after cancer diagnosis were compared by mixed models.

**Results:**

For patients with diabetes, mean HbA_1c_ during the year after lymphoma diagnosis was 6.7%. Estimated 5-year progression-free survival for patients with versus without diabetes was 63% (95% CI: 53–76%) versus 58% (95% CI: 46–71%) (p = 0.42).

**Conclusion:**

Lymphoma and its treatment did not affect glycemic control. Diabetes did not decrease lymphoma-specific survival.

## Lay abstract

This study aimed to determine the effect of diabetes on survival in patients with lymphoma and to assess whether lymphoma and its treatment affect glycemic control. We retrospectively identified 174 patients with lymphoma and matched them 1:1 (by current age, sex and age at lymphoma diagnosis) on the basis of the presence (n = 87) or absence (n = 87) of a diabetes diagnosis. Results suggest that diabetes does not decrease survival in patients with lymphoma and that lymphoma and its treatment do not affect glycemic control.

Hodgkin lymphoma and non-Hodgkin lymphoma (NHL) affect nearly 900,000 people in the USA [1] and are the most common hematologic cancers in the world [2]. With advances in treatment and early diagnosis, the survival rates for both NHL and Hodgkin lymphoma have more than doubled in the past 50 years [1]. However, approximately 20,000 Americans continue to die of lymphoma each year [1]. Understanding the relationship between lymphoma and common comorbid medical conditions remains essential for improving long-term care and survival of patients with lymphoma.

One of the most prevalent chronic conditions in the USA is diabetes, which affects more than 30 million Americans (9% of the population) [3]. It is commonly encountered in oncology practice, with more than 10% of patients with cancer having a codiagnosis of diabetes [4]. Previous studies have suggested that the correlation between diabetes and some cancers may be causal [5,6] and that diabetes may increase the risk of death in patients with cancer [7,8]. An increased risk of prostate, thyroid, lung, pancreas, liver and stomach cancers was seen among men and women with diabetes in a 2019 prospective cohort study enrolling 410,191 patients in Shanghai, China [6].

Three recent meta-analyses [9–11] reported similarly increased incidences of lymphoma among persons with diabetes. Per these meta-analyses, the relative risks of lymphoma in patients with diabetes ranged from 1.15 to 1.2 [9–11]. Proposed theories for the association between diabetes and cancer include the combined link to obesity and the proinflammatory and immunosuppressed state of diabetes [11]. However, insufficient data are available to make definitive conclusions about the relationship between diabetes and lymphoma according to prior studies [9–11].

The effect of diabetes on lymphoma survival also remains unclear. Retrospective cohort studies [12–14] have suggested that diabetes is a risk factor both for death due to NHL [12,14] and for decreased progression-free survival (PFS) [13]. However, besides these studies, few have investigated the effect of diabetes on lymphoma survival and none have discussed the effect of lymphoma on glycemic control.

Previous case–control studies of patients at a large cancer institute in the southwestern USA have shown that diabetes does not worsen survival for patients with solid organ cancers and that solid organ cancers do not worsen glycemic control. We have studied this relationship in pancreatic cancer [15], gastroesophageal cancer [16], lung cancer [17], breast cancer [18], prostate cancer [15], colorectal cancer [19] and melanoma [20]. Gastroesophageal cancer was the only cancer in which a greater risk of death was seen in patients with than without diabetes [16].

In the current study, we aimed to define baseline characteristics of patients with lymphoma and coexisting diabetes. A case–control approach was used to examine the effect of diabetes on 5-year survival of patients with lymphoma and the influence of lymphoma on glycemic control.

## Patients & methods

### Overview of the practice

This institutional review board-approved retrospective case–control study was conducted at a National Cancer Institute-accredited comprehensive cancer center in the southwestern USA which offers multidisciplinary care for more than 2700 patients with cancer every year.

### Case selection

We searched our Institutional Cancer Registry for the records of all patients with newly diagnosed lymphoma who were seen in our cancer center from 1 January 2005 through 31 December 2016. The resulting list was cross-referenced for patients seen at our institution with a diagnosis of diabetes (“International Classification of Diseases, Ninth Revision”, diagnostic code 250.00) during the same period. Patients identified with codiagnoses of lymphoma and diabetes (cases) were matched 1:1 via a greedy matching algorithm with patients with lymphoma who did not have diabetes (controls) on the basis of current age, sex and age at lymphoma diagnosis as determined from the electronic health record. The electronic health record was directly reviewed to obtain information on cancer stage, comorbid conditions, cancer therapy and diabetes duration and type of hyperglycemia management for diabetic patients. Glucose and hemoglobin A_1c_ (HbA_1c_) values at the time of lymphoma diagnosis and in subsequent years were also obtained from electronic laboratory data. Patients for whom diabetes status could not be confirmed, who were diagnosed with diabetes after lymphoma diagnosis, or who received full or partial care outside our institution were excluded from this analysis.

### Statistical analysis

For all lymphoma patients included, baseline demographics were compared between those with and without diabetes. To determine the rate of glycemic control in the study population, the percentage of patients with HbA_1c_ levels less than 7% was calculated. HbA_1c_ and glucose levels during the first year after lymphoma diagnosis were examined via mixed models and mean HbA_1c_ and glucose levels were calculated. Time (days) was considered a fixed effect and an individual-specific random effect was included for HbA_1c_. For glucose level, fixed effects included days, case or control designation, an interaction term (days × case–control designation) and patient-specific and matched pair-specific random effects.

The Kaplan–Meier method was used to estimate 5-year overall survival (OS) and PFS. OS was defined as the time from lymphoma diagnosis until death of any cause in 5 years. PFS was defined as survival duration after treatment of lymphoma without lymphoma progression. Patients without date of death documented in the electronic health record were considered censored at the last known follow-up date. To determine the effect of diabetes on OS and PFS, Cox proportional hazards regression was used, with matched pairs as the strata variable. p-values less than 0.05 were considered statistically significant. SAS version 9.4 (SAS Institute Inc., NC, USA) was used for statistical analysis.

## Results

### Patient demographics

In our cancer registry, we identified 87 patients who had both lymphoma and diabetes during the study period. These patients were matched 1:1, as described, with 87 control patients with lymphoma who did not have diabetes, for 174 patients total in the final cohort (Table 1). The mean standard deviation (SD) age at diagnosis was 68.0 (11.9) years. Most patients were White (95.4%), male (59.8%) and Medicare insured (72.4%). NHL was the most frequently seen lymphoma (171, 98.3%). Baseline characteristics were statistically similar between patients with and without diabetes, except that patients with diabetes were more likely to have a body mass index of 30.0 or higher (47.1 vs 25.3%; p = 0.005) and to be retired (56.3 vs 40.2%; p = 0.03). There was also greater use of corticosteroids among patients with diabetes (68.2 vs 41.5%; p = 0.004) (Table 1).

**Table 1.** Patient characteristics^†^.

Characteristic	Group			
	Total (N = 174)	Diabetes (n = 87)	No diabetes (n = 87)	p-value
Age at lymphoma diagnosis (years)	68.0 (11.9)	68.1 (12.0)	68.0 (11.9)	NA
Men	104 (59.8)	52 (59.8)	52 (59.8)	NA
White race	165 (95.4) (n = 173)	80 (93.0) (n = 86)	85 (97.8)	0.29
Ethnicity				NC
Hispanic	6 (3.4)	4 (4.6)	2 (2.3)	
Non-Hispanic	123 (70.7)	74 (85.1)	49 (56.3)	
Unknown	45 (25.9)	9 (10.0)	36 (41.4)	
BMI	28.7 (6.4) (n = 163)	30.3 (6.6) (n = 83)	26.9 (5.7) (n = 80)	0.003
BMI category				0.005
<25.0	62 (35.6)	23 (26.4)	39 (44.8)	
25.0–29.9	49 (28.2)	23 (26.4)	26 (29.9)	
≥30.0	63 (36.2)	41 (47.1)	22 (25.3)	
Married at time of lymphoma diagnosis	139 (80.3) (n = 173)	73 (83.9)	66 (76.7) (n = 86)	0.79
Payer type at time of lymphoma diagnosis				0.12
Medicare	126 (72.4)	64 (73.6)	62 (71.3)	
Insurance	45 (25.9)	23 (26.4)	22 (25.3)	
Self-pay	3 (1.7)	0 (0.0)	3 (3.4)	
Any alcohol use at time of lymphoma diagnosis				0.09
Yes	88 (50.6)	38 (43.7)	50 (57.5)	
No	80 (46.0)	46 (52.9)	34 (39.1)	
Unknown	6 (3.4)	3 (3.4)	3 (3.4)	
Smoking status at time of lymphoma diagnosis				0.66
Never	75 (43.1)	35 (40.2)	40 (46.0)	
Former	82 (47.1)	44 (50.6)	38 (43.7)	
Current	9 (5.2)	3 (3.4)	6 (6.9)	
Unknown	8 (4.6)	5 (5.7)	3 (3.4)	
Employment status at time of lymphoma diagnosis				0.03
Employed	52 (29.9)	25 (28.7)	27 (31.0)	
Unemployed	10 (5.7)	5 (5.7)	5 (5.7)	
Retired	84 (48.3)	49 (56.3)	35 (40.2)	
Unknown	28 (16.1)	8 (9.2)	20 (23.0)	
ECOG PS at time of lymphoma diagnosis	(n = 172)	(n = 86)	(n = 86)	0.18
0	49 (28.5)	32 (37.2)	17 (19.8)	
1	99 (57.6)	47 (54.7)	52 (60.5)	
2	16 (9.3)	5 (5.8)	11 (12.8)	
3	8 (4.7)	2 (2.3)	6 (7.0)	
Use of corticosteroids	92 (55.1) (n = 167)	58 (68.2) (n = 85)	34 (41.5) (n = 82)	0.004

† Values are mean (SD) or number of patients (%).

ECOG PS: Eastern Cooperative Oncology Group performance status; NA: Not applicable, matched variable; NC: Not calculated.

### Diabetes treatment characteristics

Case patients were confirmed to have diabetes either diagnosed before (n = 83) or newly diagnosed concurrently with (n = 4) their lymphoma diagnosis. Median duration of diabetes before lymphoma diagnosis was 8 (0–57) years (Table 2). Diabetic therapy varied. Most patients managed their diabetes with oral glycemic agents (49%) at the time of their lymphoma diagnosis and 26% were using insulin or insulin in combination with oral glycemic agents; 28% of patients managed diabetes with diet alone. For most patients with diabetes (n = 59, 68%), the method of hyperglycemic therapy did not change substantially 1 year after lymphoma diagnosis. However, insulin use increased from 26% of patients at the time of lymphoma diagnosis to 35% during the year after diagnosis (Table 2).

**Table 2.** Diabetes treatment and glycemic control in patients with lymphoma and diabetes.

Characteristic	Value (n = 87)^†^
Time since diabetes diagnosis (years)	8 (0–57)
Diabetes therapy at lymphoma diagnosis	
Diet	24 (28)
Oral	36 (41)
Insulin	16 (18)
Oral + insulin	7 (8)
Unknown	4 (5)
Insulin use	
At lymphoma diagnosis	23 (26)
Within 1 year after lymphoma diagnosis	30 (35)
HbA_1c_ 1 year after lymphoma diagnosis	(n = 51)
<7.0%	31 (61)
≥7.0%	20 (39)

† Values are number of patients (%) or median (range).

HbA_1c_: Hemoglobin A_1c_.

### Lymphoma effect on diabetes & metabolic control

**Figure 1.**
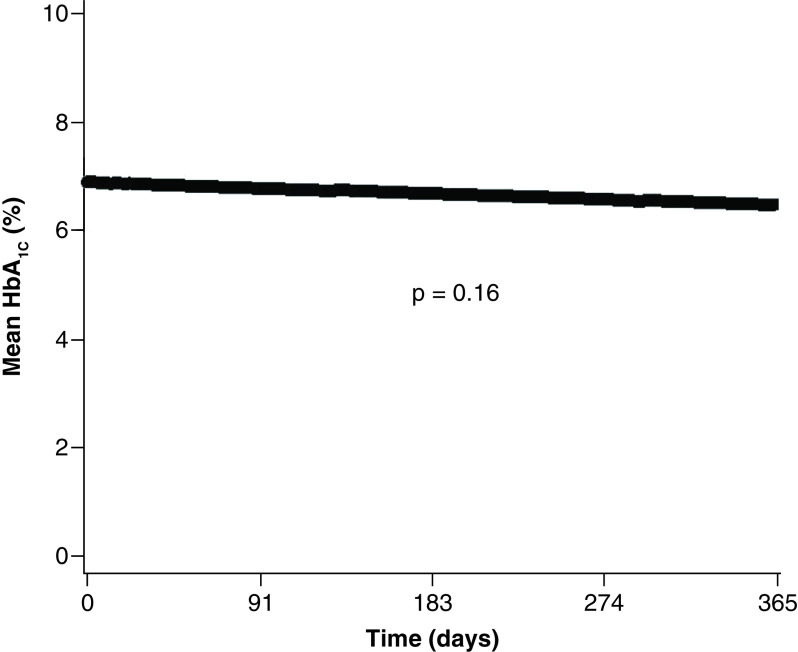
Mean hemoglobin A_1c_ values within 1 year after lymphoma diagnosis in patients with diabetes (n = 51). HbA_1c_: Hemoglobin A_1c_.

**Figure 2.**
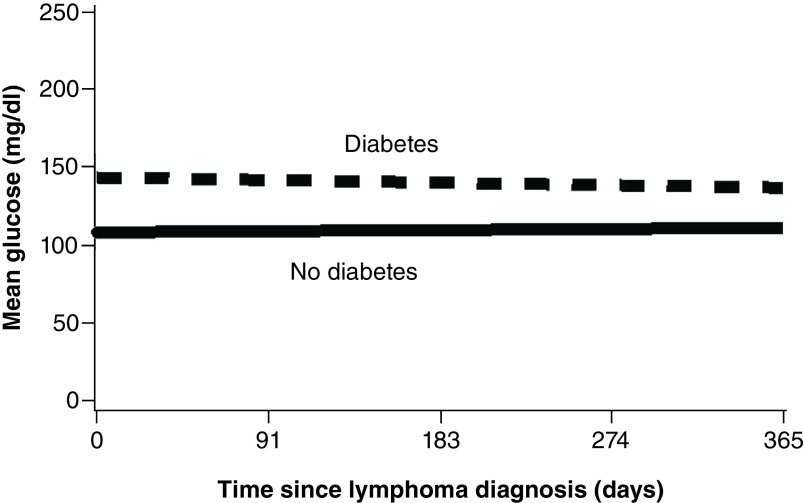
Mean glucose levels within 1 year after lymphoma diagnosis for both groups (n = 141). By mixed model, p-values were as follows: days, p = 0.75; diabetes, p < 0.001; days × diabetes, p = 0.37.

HbA_1c_ data 1 year after lymphoma diagnosis was available for 51 patients with diabetes. The estimated mean HbA_1c_ value for these patients at the time of lymphoma diagnosis was 6.9% (95% CI: 6.7–7.1%) and was 6.7% during the year after diagnosis. The majority of patients (61%) had an HbA_1c_ value less than 7.0% 1 year after lymphoma diagnosis (Table 2). HbA_1c_ values did not change significantly over time in these patients with diabetes (mixed model, days, p = 0.16) (Figure 1). Glycemic control was analyzed for patients with and without diabetes. Mean (SD) fasting glucose value was significantly higher for diabetic patients (n = 77) than for nondiabetic patients (n = 64) (142 [36] vs 108 [15] mg/dl; p < 0.001). There was no interaction effect (p = 0.37) nor a decrease over time in glucose level (p = 0.75) (Figure 2).

### Diabetes effect on lymphoma survival

**Figure 3.**
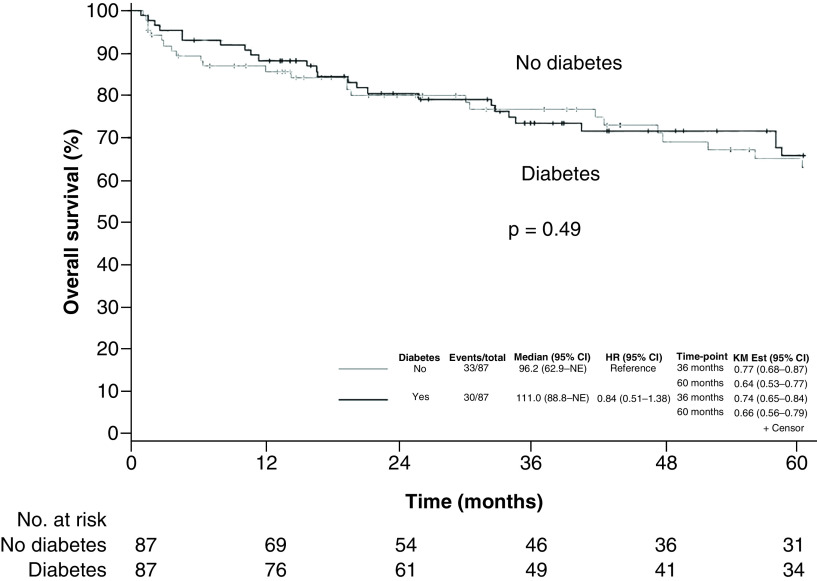
Overall survival. Kaplan–Meier curves estimating overall survival in patients with lymphoma stratified by presence or absence of diabetes. Tick marks indicate censored patients. HR: Hazard ratio; KM Est: Kaplan–Meier estimate; NE: Not estimated.

**Figure 4.**
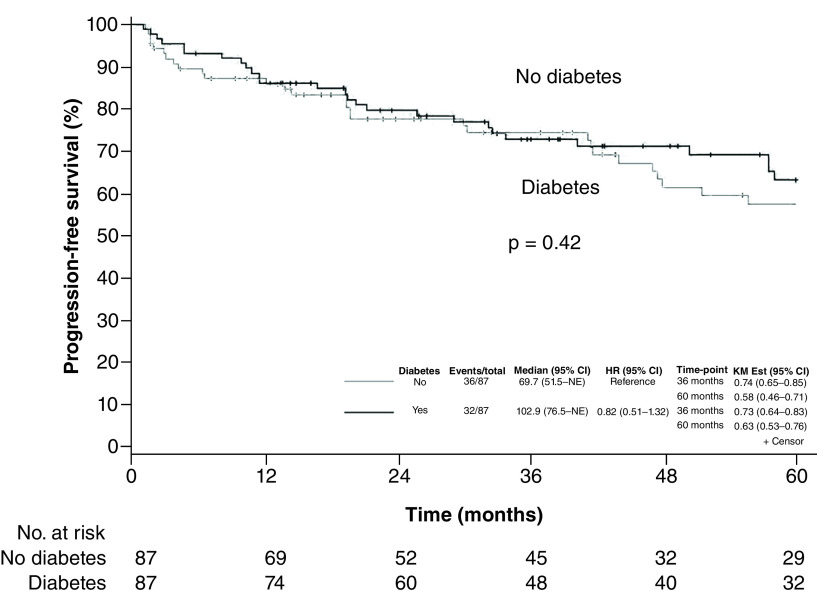
Progression-free survival. Kaplan–Meier curves estimating progression-free survival in patients with lymphoma stratified by presence or absence of diabetes. Tick marks indicate censored patients. HR: Hazard ration; KM Est: Kaplan–Meier estimate; NE: Not estimated.

The 5-year OS was 66% (95% CI: 56–79%) for patients with diabetes versus 64% (95% CI: 53–77%) for nondiabetic patients (Figure 3). When we compared matched pairs, the hazard ratio for death in the diabetic group was 0.63 (95% CI: 0.34–1.16; p = 0.14). Diabetic patients had an estimated 5-year PFS of 63% (95% CI: 53–76%) versus 58% (95% CI: 46–71%) for nondiabetic patients (Figure 4). The hazard ratio for lymphoma progression among diabetic patients was 0.66 (95% CI: 0.37–1.17; p = 0.15).

## Discussion

Because of improvements in survival for patients with lymphoma, clinicians in oncology practice are more likely to see patients who have comorbid conditions such as diabetes. In this analysis, we used matched cases and controls to investigate how diabetes affects lymphoma survival and how lymphoma affects glycemic control. Our findings suggest that diabetes does not affect the 5-year survival of patients with lymphoma. Also, lymphoma does not affect glycemic control in the year after lymphoma diagnosis.

These findings are in contrast to prior studies of interest in which patients with lymphoma who have diabetes have shown higher mortality rates and worse OS than nondiabetic controls [7,12]. A 2017 meta-analysis of 19 Asian cohort studies investigated the effect of diabetes on lymphoma survival. The hazard ratio for death among patients with lymphoma with diabetes compared with nondiabetic controls was 1.39 (95% CI: 1.04–1.86). The meta-analysis of cohort studies included 658,611 patients from East Asia and 112,686 patients from South Asia [7]. Because of interethnic differences [21], it is reasonable to assume that the results of that study may not be generalizable to the American population overall. The younger average age of participants in the meta-analysis above [7] compared with our study population (54 vs 68 years) may also account for the differences in results.

In a 2012 prospective cohort study of age- and sex-specific mortality rates in patients with lymphoma in Taiwan, age significantly influenced the increased mortality rate conferred by diabetes for patients with lymphoma [14]. The increased risk of death for patients with diabetes was greatest in patients aged 45–54 years and decreased for ages older than 65 years [14]. In contrast, another case–control study in Taiwan enrolling 242 patients with NHL suggested that the effect of preexisting diabetes on cumulative survival of patients with NHL may vary with time [12]. Preexisting diabetes did not predict decreased survival until 30 months of follow-up [12]. With our smaller sample size, follow-up time longer than 5 years may have been required to see any effect of diabetes.

The current study used a case–control design to better understand the effect of diabetes on lymphoma survival in a US population within the US healthcare system. Our findings are consistent with those of multiple case–control studies of interest from our institution – a large cancer center in the southwestern USA – that have explored the effect of diabetes on solid organ cancers and vice versa [15,17–20]. In previous investigations, diabetes was not associated with increased mortality rates for many solid organ cancers or melanoma [15,17–20]. To our knowledge, our current study is the first to evaluate the effect of lymphoma on glycemic control. Like previous studies from our institution that investigated the effect of solid organ cancers on diabetes, this study showed that lymphoma did not affect glycemic control during 1 year of follow-up.

This finding may suggest that it may not be necessary to adjust cancer treatments for fear of worsening diabetes. A 2015 systematic review regarding the effect of comorbid conditions on lymphoma reported increased mortality rates for patients with diabetes versus nondiabetic controls, possibly because diabetes resulted in decreased treatment efficacy [22]. Patients with lymphoma plus diabetes may encounter more dose adjustments in chemotherapy, delays between treatment cycles and fewer cycles of chemotherapy compared with those with lymphoma who do not have diabetes [23]. The risk of infection, hospitalization or decrease or discontinuation of chemotherapy [23] increases for patients with poor glycemic control at the start of lymphoma treatment. This may explain the higher mortality rates associated with lymphoma in patients with diabetes reported in other studies. In the current study, lymphoma patients with diabetes had higher mean glucose levels than those without diabetes, but it is not clear from our chart review whether this delayed or influenced therapy in this population.

Despite a greater use of corticosteroids in the diabetes group in our study, mean blood glucose or HbA_1c_ values did not increase significantly during the first year after lymphoma diagnosis. It might be speculated that our population of patients with relatively well-controlled diabetes (majority with HbA_1c_ <7%) may have had few diabetes-related complications or treatment adjustments. The patients with diabetes in this study may have represented a cohort that was compliant with their diabetes medications and other diabetes self-management skills (e.g., diet, glucose monitoring) and who continued to be diligent throughout their lymphoma treatment course. Acute exacerbations of hyperglycemia most likely did occur with glucocorticoid therapy. However, patients and physicians may be reassured that glucose control showed no chronic worsening at least over the first year of lymphoma treatment. Nearly 50% of our patients with diabetes were managing their diabetes with oral hypoglycemic agents. On a molecular basis, oral hypoglycemic agents such as metformin may have activity on adenosine monophosphate-activated protein kinase, a critical factor in cell sensitivity to anticancer drugs, as shown in a previous study [24]. That study suggested that metformin may inhibit the growth of B- and T-cell lymphomas and improve the response of lymphoma to drugs such as doxorubicin and temsirolimus. This may explain why diabetes did not worsen lymphoma survival. Additionally, because synergistic drugs potentiate each other’s effects, standard NHL therapy may enhance the effects of oral hypoglycemic agents, helping to maintain glycemic control. Further molecular investigation into the effects of oral hypoglycemic agents on standard lymphoma therapy and vice versa, should be explored.

Our findings are subject to several limitations. This was a retrospective study and our analysis is based on data available in the electronic health record. Glucose levels were obtained at provider follow-up visits or during hospitalization. Although glucose levels in these settings are primarily taken under fasting conditions, it is possible that some were not. This study was performed at a single center and the patient population studied was primarily White, elderly and Medicare insured. Thus, our results have limited generalizability to other races, ages and socioeconomic groups. The sample size was small and primary end points were analyzed at short follow-up times of 1 and 5 years after lymphoma diagnosis. Thus, comments on the possible long-term effects of diabetes on lymphoma and vice versa cannot be made from this study. Finally, NHL made up the primary lymphoma diagnosis in the sample population, so findings may not apply to patients with Hodgkin lymphoma.

## Conclusion

This research expands our understanding of the relationship between diabetes and hematologic cancers, like lymphoma. It is the first study to investigate the effect of lymphoma on glycemic control. It is also one of few published studies and, to our knowledge, the only US-based study that evaluated the effect of diabetes on lymphoma survival. In this analysis, diabetes did not affect short-term lymphoma survival and lymphoma did not affect glycemic control.

## Future perspective

The results of this study may reassure providers that diabetes does not affect OS of patients with lymphoma and that treatment of lymphoma does not affect short-term glycemic control in patients with diabetes. Future studies employing larger sample sizes and longer follow-up times are needed to provide optimal care for patients with codiagnoses of lymphoma and diabetes. Also, the effect of diabetes on other hematologic cancers should continue to be explored.

**Executive summary**

- The effect of lymphoma or its treatment on diabetes and the effect of diabetes on lymphoma survival has been an area of uncertainty.
- Analyzed patients with diabetes and lymphoma were more likely to be obese than their nondiabetic counterparts (p = 0.003) and to have higher use of corticosteroids (p = 0.004).
- Patients with diabetes had higher mean glucose levels than patients without diabetes, but the mean glucose level did not change significantly for either group in the year after lymphoma diagnosis.
- Among patients with diabetes, mean (standard deviation) hemoglobin A_1c_ 1 year after lymphoma diagnosis was 6.7% (1.1%) and did not significantly change.
- The 5-year overall survival was 66% (95% CI: 56–79%) for patients with diabetes versus 64% (95% CI: 53–77%) for nondiabetic patients (hazard ratio: 0.63; 95% CI: 0.34–1.16; p = 0.14).
- The 5-year progression-free survival was 63% (95% CI: 53–76%) for patients with diabetes versus 58% (95% CI: 46–71%) for nondiabetic patients (hazard ratio: 0.66; 95% CI: 0.37–1.17; p = 0.15).
